# Airway Epithelial Repair by a Prebiotic Mannan Derived from *Saccharomyces cerevisiae*

**DOI:** 10.1155/2017/8903982

**Published:** 2017-07-09

**Authors:** Christie F. Michael, Christopher M. Waters, Kim S. LeMessurier, Amali E. Samarasinghe, Chi Y. Song, Kafait U. Malik, D. Betty Lew

**Affiliations:** ^1^Department of Pediatrics, University of Tennessee Health Science Center (UTHSC), Memphis, TN 38103, USA; ^2^Children's Foundation Research Institute, Le Bonheur Children's Hospital, Memphis, TN 38103, USA; ^3^Department of Physiology, University of Tennessee Health Science Center (UTHSC), Memphis, TN 38163, USA; ^4^Department of Pharmacology, University of Tennessee Health Science Center (UTHSC), Memphis, TN 38163, USA

## Abstract

In asthmatic airways, repeated epithelial damage and repair occur. No current therapy directly targets this process. We aimed to determine the effects of mannan derived from *S. cerevisiae* (SC-MN) on airway epithelial wound repair, in vitro. The presence of functional mannose receptors in bronchial epithelial cells was shown by endocytosis of colloidal gold-Man BSA via clathrin-coated pits in 16HBE cells. In primary normal human bronchial epithelial cells (NHBEC), SC-MN significantly facilitated wound closure. Treatment with SC-MN stimulated cell spreading as indicated by a significant increase in the average lamellipodial width of wound edge 16HBE cells. In addition, NHBEC treated with SC-MN showed increased expression and activation of Krüppel-like factors (KLFs) 4 and 5, transcription factors important in epithelial cell survival and regulation of epithelial-mesenchymal transition. We conclude that SC-MN facilitates wound repair in human bronchial epithelium, involving mannose receptors.

## 1. Introduction

The hallmarks of asthma exacerbation include inflammation, denudation of the bronchial epithelial barrier, and focal airway damage that is evident on bronchial biopsy even in mild asthmatics [1, 2]. The consequences of repeated epithelial damage and repair are not completely understood, but the disrupted epithelial barrier and epithelial dysfunction are crucial in the development and maintenance of asthma symptoms [3]. This process, triggered by a variety of factors such as ozone [4], radical-containing particles [5], viral infections [6], cigarette smoke [7], and allergens, can ultimately contribute to airway remodeling [3, 7]. Inhaled corticosteroids have been shown to prevent epithelial damage in asthmatics [8]. However, this is likely an indirect result of anti-inflammatory effects rather than a direct protective or repair function. A report by Dorscheid et al. demonstrated that steroid treatment prior to wounding airway epithelial cells in vitro resulted in impairment of both epithelial cell migration and wound closure and induced apoptosis of inflammatory cells and epithelial cells [9]. Indeed, none of the current acute or control therapies for asthma specifically target epithelial integrity or repair. Resolution of airway hyperreactivity (AHR) has been reported to correlate with restoration of the epithelium [1, 10], whereas AHR may persist after resolution of cellular inflammation in human asthma [11].

Moreover, anti-inflammatory therapy alone has not been shown to halt disease progression in asthma [12]. Thus, a therapy that could promote epithelial repair should provide adjunctive therapeutic benefit for asthma.

Mannan, polymannose glycoprotein in the cell wall of various bacterial and fungal species, may have species-specific structure and lipid modifications that regulate the degree of dendritic cell mannose receptor clustering, differential cytokine profile, and subsequent effector functions [13]. Mannan derived from mycobacteria has been shown to suppress allergic airway disease in a murine-allergic asthma model [14]. On the other hand, mannan derived from pathogenic fungi is likely to induce Th17 inflammatory cytokines [15]. This is in contrast to mannan derived from the outer wall of the commensal yeast *Saccharomyces cerevisiae* (SC-MN), which does not stimulate Th17 cytokines [15]. However, direct effects of mannan, from any source, on airway epithelium, are unknown. In this study, we show that human bronchial epithelial cells (HBEC) express a functional mannose receptor. We also present evidence that SC-MN facilitates wound healing of bronchial epithelium in vitro, complementary to its antiproliferative effect in airway smooth-muscle cells that we have previously described [16, 17]. At a transcriptional level, we present evidence that SC-MN stimulates expression and activation of Krüppel-like factors (KLFs) 4 and 5, key transcription factors for epithelial cell differentiation, survival, and proliferation [18].

## 2. Methods

### 2.1. Cell Cultures

Either primary normal human bronchial epithelial cells (NHBEC, Lonza, Walkersville, MD) or an immortalized line of human bronchial epithelial cells, 16HBE (kindly provided by Dr. Gruenert, California Pacific Institute) was cultured either in 6-well plates, in 24-well plates on Transwell inserts with 0.45 *μ*m pores at a liquid/liquid interface, or on 18 mm glass coverslips in 12-well plates to confluence at 37°C and 5% CO_2_. The NHBEC were cultured in basal epithelial growth media (BEM) supplemented with BulletKit™ (Lonza) at 37°C and 5% CO_2_.

### 2.2. Mannose Uptake Studies

16HBE cells were grown in on a nylon sheath in Transwell culture plates and were incubated with gold-labeled mannose-bovine serum albumin (10 nm) (ManBSA) or gold-BSA (control) (EY Labs Inc., San Mateo, CA) for 30 min and processed for electron microscopy as we previously described [17].

### 2.3. Wound Repair Assay

Using a pipette tip (5–200 *μ*l capacity), mechanical wounds were inflicted across the diameter of the wells. Wounded cells were cultured with medium containing 10% fetal bovine serum (HyClone, Logan, UT) and 1% antibiotic/antimycotic solution (Sigma, St. Louis, MO). Cells were incubated with vehicle control (saline, final concentration: 0.005%), SC-MN (0.5–1.0 mg/ml, approximately 14–28 *μ*M) (Sigma, prepared for patented use for asthma therapeutic), budesonide (10^−7^ – 10^−6^ M), heat-inactivated (50°C, 3 hr) beta-hexosaminidase A (HI-Hex A, endogenous ligand for mannose receptor, 50 nM, microdialyzed to remove ammonium sulfate and stored in 5% glycerin-containing phosphate buffer, Sigma), or a combination of SC-MN and HI-Hex A. Endotoxin levels of these agents were less than 2 EU/ml. Primary NHBEC were fixed and stained with crystal violet for overall assessment of wound closure under phase microscopy. Analysis of the perimeter area of the remaining wound in each image was performed using *ImageJ* software (National Institutes of Health, Bethesda, MD). For lamellipodial analysis, cells were fixed with 3.7% formaldehyde and permeabilized with 0.1% Triton X, followed by staining with rhodamine phalloidin (Molecular Probes, Eugene, OR) according to the manufacturer's protocol and then images were acquired using fluorescence microscopy [19]. A number of total wound edge cells expressing lamellipodia were counted, and measurements were made using MetaMorph imaging software (version 4.6; Universal Imaging, West Chester, PA).

### 2.4. Western Blot Analysis

NHBE cell lysates were analyzed for Krüppel-like factors 4 and 5 (KLF4 and KLF5) and phospho-KLF4 and phosphor-KLF5 by Western blot. Protein concentration was determined by Bradford method (Thermo Scientific, Rockford, IL) and 10 *μ*g was loaded per lane for SDS-polyacrylamide gel electrophoresis (8%). Blots of protein bands were probed with primary (KLF4 (rb, 1 : 500 dilution, EMD Millipore, Billerica, MA); p-KLF4 (Ser245) (rb, 1 : 500, Abgent, San Diego, CA); KLF5 (rb, 1 : 500, AVIVA System Biology, San Diego, CA); and p-KLF5 (Ser311) (rb, 1 : 500, Bioss Inc., Woburn, MA; beta-actin) (mo, 1 : 1000, Santa Cruz, Dallas, TX)) and appropriate secondary antibodies and intensity of the bands was measured with *ImageJ* 1.42 software.

### 2.5. Statistical Analysis

Data are presented as median +/− SD. Data were analyzed using the nonparametric Kruskal-Wallis one-way analysis of variance test (Graphpad Prism 6, La Jolla, CA). *p* values less than 0.05 were considered significant.

## 3. Results

### 3.1. Presence of Functional Mannose Receptors in Human Epithelial Cells

**Figure 1:**
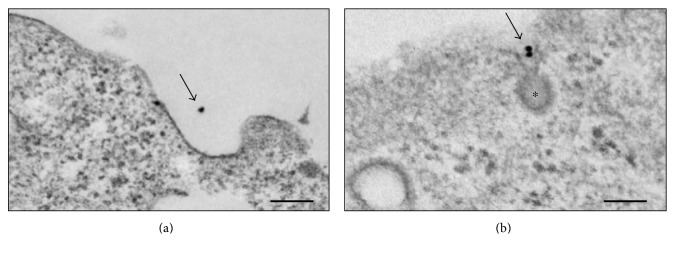
Endocytosis of colloidal gold-mannose bovine serum albumin (ManBSA) via clathrin-coated pits in 16HBEC. (a) Gold-BSA; (b) gold-ManBSA. ^∗^Clathrin-coated pit. Arrows: gold particles. Electron microscopy scale bar: 100 nm.

To assess whether bronchial epithelial cells express mannose receptors, 16HBE cells grown on 24-well Transwell plate inserts were exposed to colloidal gold-Man BSA (10 nm particle size) for 30 min at 37°C. In control experiments, the cells were treated with gold-BSA (Figure 1(a)). Electron microscopic examination of the cells showed gold particles being endocytosed via clathrin-coated pits in 16HBE cells, indicating the presence of functional mannose receptors (Figure 1(b)) [17].

### 3.2. Facilitation of Repair by SC-MN on Mechanically Inflicted Wounds in NHBEC

**Figure 2:**
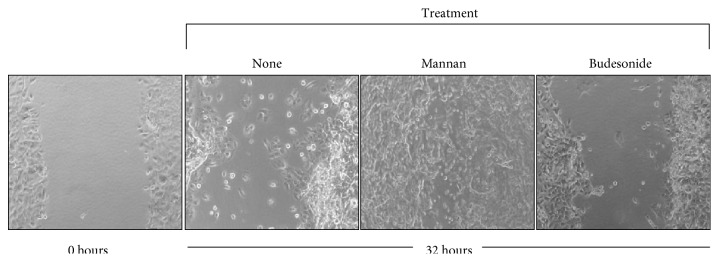
SC-MN facilitates wound closure in NHBEC. Mechanically injured NHBEC were treated with saline (none), SC-MN (1 mg/ml), or budesonide (10^−7^ M). Final magnification: 50x.

**Figure 3:**
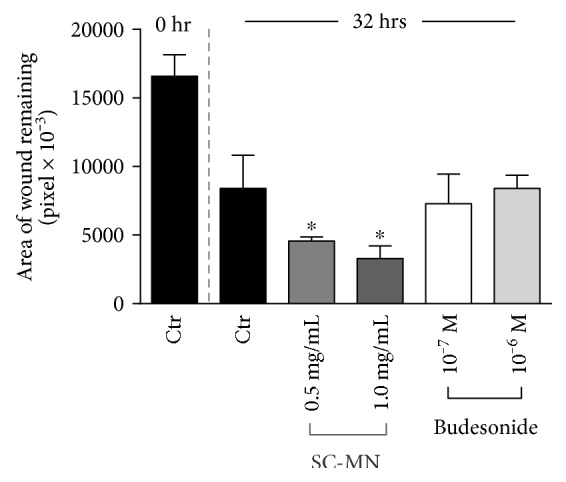
SC-MN's beneficial effect on mechanical wound closure in NHBEC. SC-MN at 0.5 and 1.0 mg/ml concentration significantly facilitated wound closure compared to the control- (saline-) treated cultures. Budesonide had no such effect. ^∗^*p* < 0.05.

Mannan from *S. cerevisiae* enhanced wound closure in primary NHBEC. SC-MN-treated cells (1 mg/ml) showed accelerated wound closure compared to the control cultures and budesonide-treated cells (10^−7^ M) (Figure 2, images acquired at the 32 hr time point). Budesonide was used for comparison because this inhaled corticosteroid is frequently used to treat asthma in young children. The average number of wound edge cells expressing lamellipodial extensions analyzed at the 24 hr time point under high-power magnification was 5/14 (36%) for untreated controls, 11/11 (100%) for mannan-treated cultures, and 8/14 (57%) for budesonide-treated cultures. Measurements of the remaining wound area at the 32 hr time point are shown in Figure 3. SC-MN (0.5 and 1.0 mg/ml) significantly facilitated wound closure compared to the control cultures in contrast to the lack of beneficial effect by budesonide treatment.

### 3.3. Facilitation of Repair by SC-MN on Mechanically Inflicted Wounds in 16HBEC

**Figure 4:**
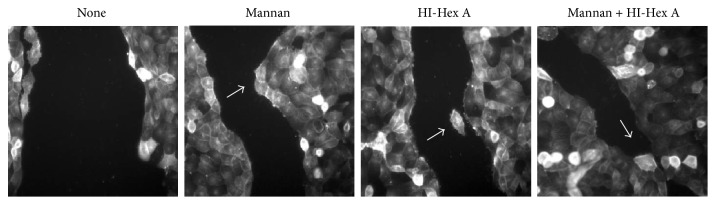
SC-MN and heat-inactivated (HI) Hex A facilitate wound closure in 16HBE cells. Digital images of cells after 17 hrs incubation (final magnification: 300x). Arrows: evagination of migrating wound edges.

**Figure 5:**
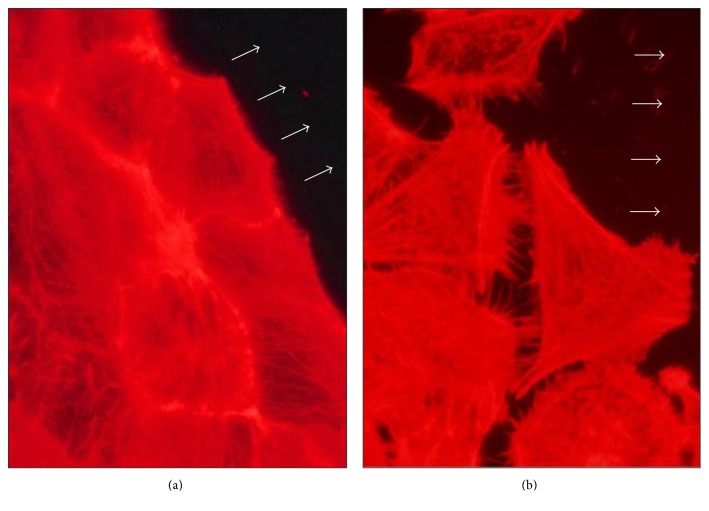
Effect of SC-MN on 16HBEC migration. Mechanically injured cells on glass coverslips were treated with saline (a) or SC-MN (1 mg/ml) (b) for 13 hrs. Arrows: the direction of cell migration (final magnification: 500x).

Similar results were obtained in a faster growing 16HBE cells (Figure 3) when wound closure was examined over time. There was no significant difference in closure at 8 hrs, but by 13 hrs, differences became evident. At 17 hours, the SC-MN- or HI-Hex A-treated wounds had 30% or less wound area remaining, versus the 50% or greater remaining for control cultures, respectively (data not shown). Combining SC-MN (1 mg/ml) and HI-Hex A (50 nM) appeared to maximize wound closure (Figure 4). Accelerated 16HBE cell spreading and migration were apparent at the cellular level as early as 8 hrs in all three conditions (mannan treated, HI-Hex A treated, and a combination of these agents), compared to those of control cells.  Figure 5 shows images of rhodamine-phalloidin-stained actin. Average lamellipodial width of wound edge cells at 13 hours after wounding was significantly increased in SC-MN-treated (4.47+/− 0.90 microns, *n* = 10) versus control 16HBE cells (1.57+/− 0.59 microns, *n* = 6) (*p* < 0.05), indicating increased spreading and enhanced migration. The overall appearance of the wound edge cells in the HI-Hex A or SC-MN-treated groups was consistent with an active healing process not only by lamellipodial extensions but also by directionality toward the opposing wound edge (Figure 5).

### 3.4. Effect of SC-MN on KLF4 and KLF5 Expression and Activation in NHBE Cells

**Figure 6:**
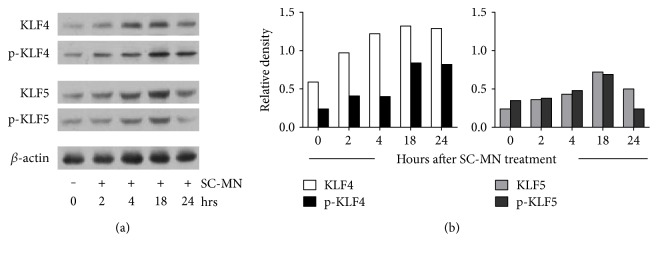
Expression and activation of KLF4 and KLF5 by SC-MN in NHBEC. (a) Phosphorylated forms indicate protein activation. (b) Density of each protein relative to *β*-actin. Data are representative of two independent experiments.

Although mannose receptor (MR) activation in airway smooth muscle cells is known to involve signal transduction pathways including transient elevation of cAMP [20], PKC activation [21], and ERK1/2 activation [22], the mechanism by which SC-MN affects epithelial repair is unknown. To gain insight into postreceptor mechanisms at the transcriptional level, we assessed whether the reparative effects of SC-MN on airway epithelial cells involved transcriptional factors KLF4 or KLF5, which are known to be associated with cell differentiation, survival, and proliferation [18]. Mannan from *S. cerevisiae* (1 mg/ml) stimulated the expression and phosphorylation of KLF4 and KLF5 (Figure 6) at 2–18 hr time points in NHBEC.

## 4. Discussion

We present evidence that a functional MR family member is expressed in human bronchial epithelial cells; this is supported by endocytosis of mannosylated neoglycoprotein in clathrin-coated pits. This adds to the list of MR in the epithelium: retinal pigment epithelial cells [23] and nasal polyposis [24]. In search of a therapeutic that can directly impact the epithelial damage occurring in asthma, we have discovered that SC-MN facilitates healing of human bronchial epithelial wounds in vitro. The healing effect of SC-MN on primary NHBEC was significant compared to that of the control cells and contrasted to budesonide (glucocorticosteroids) treatment which did not improve wound healing. Asthma research on beta-hexosaminidases (Hex A and Hex B), endogenous enzymes, and known ligands for mannose receptors, has been hampered by the lack of highly purified enzyme (the preparation used for our previous work was 150–170 times more pure compared to the commercially available beta-hexosaminidases) [16]. Nevertheless, heat-inactivated Hex A, produced a similar healing effect on airway epithelium, further supporting the presence of a mannose receptor in human bronchial epithelium.

Epithelial repair mechanisms involve cell migration and proliferation, promoted by multiple growth factors, chemokines (MCP-1), cytokines (IL-1*β*, IL-2, IL-4, and IL-13), and prostaglandins (PGE2) that work in coordination with integrins and other matrix materials (fibronectin, collagen, and laminin) [25]. Treatment of bronchial epithelial cells with SC-MN does not produce the abovementioned cytokines (preliminary unpublished data). Many cellular molecular factors, such as Sonic hedgehog, Rho GTPases, MAP kinase pathways, STAT3, and Wnt [25], contribute to epithelial cell migration and proliferation. In addition to promoting wound closure, SC-MN appears capable of initiating directional migration of human bronchial epithelial cells. However, the specific mechanisms of how SC-MN directs epithelial migration and proliferation downstream of MR are unknown.

In order to determine the underlying mechanisms of airway epithelial wound healing by SC-MN, KLF4 and KLF5 expression were determined. These transcription factors are known to be associated with differentiation, survival, and cell proliferation. Within 2 hrs of cell exposure to SC-MN, KLF4 expression and activation (by phosphorylation) were apparent. This association of KLF4 expression and activation is likely a contributing factor to directional migration of epithelial cells during wound closure. SC-MN also induced KLF5 expression and activation in NHBEC at 2–18 hrs (peak at 18 hrs), and KLF5 is known to inhibit epithelial mesenchymal transition by microRNA 200 transcription [26].

There are limitations of this study in respect to cell types and culture system. Primary human bronchial cells from asthmatic patients would have provided further insight to the beneficial effect of mannan in asthma; however, they are extremely difficult to acquire. In addition, the culture of NHBEC in an air-liquid interface model could elucidate mannan's effect on differentiated cells. Regarding the mechanisms of SC-MN's action, it is possible that mannan may work through alternative mechanisms in epithelial cells other than a receptor-mediated phenomenon. For example, it may act as a barrier protection as it does in yeast. Another possibility is that endotoxin of SC-MN, even though the concentration is minimal, might have had some influence in epithelial cells.

The mannose receptor and other endogenous glycan-binding proteins, implicated in both innate and adaptive immunity, represent a largely unexplored opportunity to modulate immune responses. Glycoprotein endogenous ligands for MR (*β*-Hex A and B, aka NAGA (N-Acetyl-*β*, D-Glucosaminidase), NAG, and NAGase) are elevated in bronchoalveolar lavage fluids of guinea pigs exposed to ozone [4] and in serum of asthmatic patients, more so in severe asthmatics [27]. Moreover, gene variants of glycoprotein and resulting abnormal serum levels of the respective glycoproteins are associated with asthma [28]. Although the specific subtype of mannose receptor expressed in airway bronchial epithelial cells is yet to be determined, sufficient evidence exists to indicate the presence of a functional receptor in the mannose family. SC-MN is an appealing agent with therapeutic potential for repairing wounded bronchial epithelium in addition to its already known effect of antimitogenic effect in airway smooth muscle cells [16]. We conclude that SC-MN mediates a direct healing effect on human bronchial epithelial cell wounds, in part, by engagement of a mannose receptor. Intricate regulation by SC-MN at the transcriptional level involving KLF4 and KLF5 and their posttranslational modifications (phosphorylation, acetylation, and methylation) needs further investigation.

## Abbreviations

EMT: Epithelial-mesenchymal transition
NHBEC: Normal human bronchial epithelial cells
KLF: Krüppel-like factor
ManBSA: Mannosylated bovine serum albumin
OVA: Ovalbumin
SC-MN: *Saccharomyces cerevisiae* mannan
Hex A: *β*-Hexosaminidase A
HI: Heat inactivated.
